# 

**Published:** 1896-02

**Authors:** 


### BOOKS RECEIVED.

---

Report of the Commissioner of Education for the year 1892-93. Volume I. Containing Parts I. and II. Washington : Government Printing Office. 1895.

Transactions of the Medical Society of the State of North Carolina. Forty-second annual meeting, held at Goldsboro, N. C., May 14, 15 and 16, 1895. Wilmington, N. C.: Legwin Brothers. 1895.

Transactions of the American Surgical Association. Volume XIII. Edited by De Forest Willard, A. M., Ph. D., M. D., Recorder of the Association. Philadelphia : Printed for the Association and for sale by William J. Dornan. 1895.

Diet in Sickness and Health. By Mrs. Ernest Hart. Formerly Student of the Faculty of Medicine of Paris, and of the London School of Medicine for Women. With an introduction by Sir Henry Thompson, F. R. C. S , M. B., London. Pp. xii.—219. Philadelphia : W. B. Saunders, 925 Walnut Street. 1895.

Therapeutics in Infancy and Childhood. By A. Jacobi, M. D., Clinical Professor of the Diseases of Children in the College of Physicians and Surgeons (Columbia University), New York ; President of the

Association of American Physicians; late President of the New York Academy of Medicine and of the Medical Society of the State of New York, etc. Small 8vo, pp. 518. Philadelphia: J. B. Lippincott Co. 1896.

An American Text-Book of Surgery, for Practitioners and Students. By Charles H. Burnett, M. D., Phineas S. Conner, M. D., Frederic S. Dennis, M. D., William W. Keen, M. D., Charles B. Nancrede, M. D., Roswell Park, M. D., Lewis S. Pilcher, M. D., Nicholas Senn, M. D., Francis J. Shepherd, M. D., Lewis A. Stimson, M. D., William Thompson, M. D., J. Collins Warren, M. D., and J. William White, M. D. Edited by William W. Keen, M. D., LL. D., and J. William White, M. D., Ph. D. Second edition, carefully revised. Illustrated. Imperial 8vo, pp. xiv.—1248. Price, $7.00 cloth; $8.00 sheep and $9.00 half-Russia. Philadelphia: W. B. Saunders, 925 Walnut Street. 1895.

The Functional Examination of the Eye. By J. Herbert Claiborne, Jr., M. D., Adjunct Professor of Ophthalmology in the New York Polyclinic; Instructor in Ophthalmology, College of Physicians and Surgeons, New York; Assistant Surgeon to the New Amsterdam Eye and Ear Hospital; Author of Theory and Practice of the Ophthalmoscope. 100 pages with twenty-one illustrations. Price, $1.00. Philadelphia: The Edwards & Docker Co. 1895.

Diphtheria and its Associates. By Lennox Browne, F. R. C. S., Ed., Senior Surgeon to the Central London Throat, Nose and Ear Hospital, etc. Octavo, pp. xii.—270. Illustrated by the author. Price, $5.00. Philadelphia: J. B. Lippincott Co. 1895.

The American Year-Book of Medicine and Surgery. Being a Yearly Digest of Scientific Progress and Authoritative Opinion in all Branches of Medicine and Surgery drawn from Journals, Monographs and Text-Books of the leading American and Foreign Authors and Investigators. Collected and arranged with Critical Editorial Comments. By J. M. Baldy, M. D., C. H. Burnett, M. D., Archibald Church, M. D., C. F. Clarke, M. D., J. Chalmers DaCosta, M. D., W. A. N. Dorland, M. D., V. P. Gibney, M. D., Homer W. Gibney, M. D., Henry A. Griffin, M. D., John Guitéras, M. D., C. A. Hamann, M. D., H. F. Hansell, M. D., W. A. Hardaway, M. D., T. M. Hardie, B. A., M. B., C. F. Hersman, M. D., B. C. Hirst, M. D., E. Fletcher Ingals, M. D., W. W. Keen, M. D., H. Leffman, M. D., V. H. Norrie, M. D., H. J. Patrick, M. D., William Pepper, M. D., D. Riesman, M. D., Louis Starr, M. D., Alfred Stengel, M. D., G. N. Stewart, M. D., and Thompson S. Westcott, M. D. Under the general editorial charge of George M. Gould, M. D. Profusely illustrated with numerous woo-dcuts in text and thirty-three handsome half-tone and colored plates. Royal 8vo, pp. vi.—1183. Price, $6.50. Philadelphia: W. B. Saunders. 1896.

